# Genetic changes in grapevine genomes after stress induced by *in vitro* cultivation, thermotherapy and virus infection, as revealed by AFLP

**DOI:** 10.1590/S1415-47572009005000079

**Published:** 2009-12-01

**Authors:** Miroslav Baránek, Jana Raddová, Bretislav Krizan, Miroslav Pidra

**Affiliations:** Mendeleum Institute, Horticulture Faculty of MUAF in Brno, LedniceCzech Republic

**Keywords:** grapevine, stress, somaclonal variation, virus infection, AFLP

## Abstract

The Amplification Fragment Length Polymorphism (AFLP) technique was employed to study genetic variations which can be induced in vines by the stress occurring during different aspects of viticulture (*in vitro* cultivation, *in vitro* thermotherapy and virus infection). Analysis of AFLP banding patterns, generated by using 15 primer combinations, pointed to negligible genetic variation among plants exposed to individual stress. The average of similarity coefficients between differently stressed plants of the cultivars Müller Thurgau and Riesling were 0.984 and 0.991, respectively, as revealed by AFLP analysis. The low incidence of observed polymorphism demonstrates the high level of genome uniformity in plants reproduced by *in vitro* micropropagation via nodes, those subjected to *in vitro* thermotherapy and virus-infected plants.

Many changes in the phenotypes and genotypes of plants after exposure to stress have been recorded (Jain, 2001; Hazarika, 2006; Saker *et al.*, 2006; Oh *et al.*, 2007). The molecular basis for this phenomenon is not precisely known, but both genetic and epigenetic mechanisms have been proposed. In some plant species, such as those of ornamental plants, such newly-induced variability can be a desirable source of novel, interesting phenotypes. On the other hand, in the case of stress arising from the manipulation of grapevine clones which may possess unique characteristics as a result of purposeful selection carried out over centuries, such changes are rather undesirable.

*In vitro* manipulation, one of the commonly used techniques in modern viticulture, represents a potential stress factor. It is used for the propagation of plant material, mainly by means of nodal cultures with apical or axillary buds. Furthermore, *in vitro* techniques are used in thermotherapy, as a procedure designed to eliminate the viruses present in infected plant material and so produce virus-free plants. Compared to conventional *in vitro* propagation, the long period of exposure to high temperatures and the necessity of manipulating apical segments containing meristem tissues could increase the probability of induced somaclonal variation occurring.

One of the most significant stress factors for plants in vineyards could also be virus infections. In monitoring the presence of the seven most wide-spread viruses in the Czech republic (which represents only a small proportion of the approximately 50 viruses that have so far been recognised in grapevines), 45% of arbitrarily selected vines were found to be infected with at least one virus (Kominek and Holleinova, 2003). The effects of virus infection on plant growth and fruit yield/quality can be widely seen in commercial vineyards. More specific studies have described changes in photosynthetic pigments and their activity (Bertamini *et al.*, 2004), transcriptome variance (Espinoza *et al.*, 2007) or polyphenols in infected plants (Tomazic *et al.*, 2003).

Little information has been provided to date about the degree of changes in regenerated grapevine somaclones or, more broadly speaking, in stressed grapevines. On the basis of ampelographic data it has been concluded that *in vitro-*regenerated somaclones are very similar but not identical (Grenan, 1984; Dami and Hughes, 1995; Gribaudo *et al.*, 2000). The few existing investigations into genome changes are mainly based on cytological measurements (Kuksova *et al.*, 1997; Leal *et al.*, 2006). Strong changes in rDNA methylation have been demonstrated by Harding *et al.* (1996) in the case of micropropagated subculture analysis, and Popescu *et al.* (2002) found polymorphism in anther-derived plantlets by using MS-AFLP and even standard AFLP techniques. To study tissue-culture induced variation, Schellenbaum *et al.* (2008) analysed 78 grapevine somaclones from somatic embryos of two distinct cultivars using SSR, AFLP and MSAP markers. They found no polymorphism between somaclones and the respective mother clones by SSR, whereas AFLP polymorphism between motherclones and somaclones was 1.3-2.8 times higher than that found between clones.

In this work, the impact of the above-mentioned stress factors on the primary structure of DNA was studied by means of the analysis of data derived from various Müller Thurgau and Riesling somaclones (Figure 1, Table 1). In so doing, standard conditions for *in vitro* propagation and thermotherapy were used. DNA extracts from the differently stressed plants (“variants”) were analysed by means of AFLP, the especially suitable method for studies where a low degree of genetic diversity is expected.

At first the vines, in a confirmed virus-free state, were selected as aboriginal mother-plants (one for Müller Thurgau, one for Riesling), and were planted in an insect-proof greenhouse in Lednice (Czech Republic). The woody stems from 5-year old plants (20 from each mother-plant) were cut into one-node pieces, planted into plastic containers with soil and numbered from 1 to 20. Some of the cuttings (nos. 8-20 for both cultivars) were inoculated with Grapevine Fanleaf virus (GFLV) via inarching with infected internodes, and subsequently tested by RT-PCR methods to confirm the presence of the GFLV virus. Cuttings which repeatedly showed a positive reaction to GFLV (tested as in MacKenzie *et al.*, 1997), as well as healthy cuttings (nos. 1-7, not inoculated), were then used for the establishment of both *in vitro* cultures and *in vitro* thermotherapy. The aim was to obtain three replicates each of the stress treatments included in this study and their various combinations (see Table 1), whereas only complete sets of variants originating from one cutting were included in the final group. The procedure for preparing individual variants is summarized in Figure 1. The final group of selected variants for analysis is listed in Table 1.

*In vitro* cultures were established using nodal segments grown on an Murashige and Skoog (MS) medium (Murashige and Skoog, 1962) containing 0.3 mg.L^-l^ 6-benzylaminopurine (BA) and 0.1 mg.L^-1^ Indole-3-acetic acid (IAA). The cultures were maintained at 23 °C with a 16/8 h cycle of light and dark. Fluorescent tubes with cool-white light were used, its Photosynthetic Photon Flux (PPF) was adjusted on 20.2 μmol.m^-2^.s^-1^. The experimental plants were transferred to a fresh medium after three weeks. Each plant was placed into a separate test tube which was then marked. Six-week old cultures were either rooted on MS media with 0.15 mg.L^-1^ 1-Naphtalene acetic acid (NAA) or exposed to *in vitro* thermotherapy.

**Figure 1:**
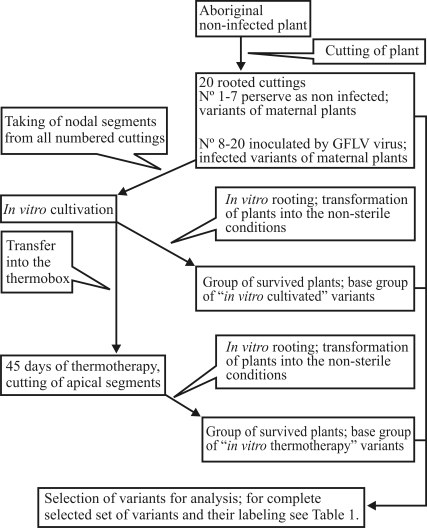
Schema of the preparation of individual variants.

For *in vitro* thermotherapy, a plants propagated on an MS medium (Murashige and Skoog, 1962) containing 0.3 mg.L^-l^ BA and 0.1 mg.L^-1^ IAA were placed into a thermobox with a photoperiod of 16 h of light and 8 of dark, at a temperature of 37 °C. Relative air humidity and light intensity in PPF were set at 80% and 22 μmol.m^-2^.s^-1^, respectively. After 45 days of thermotherapy, apical segments were sampled and placed onto the same MS medium. After two weeks, when the plants were about 15 mm high, they were transferred to rooting media MS with 0.15 mg.L^-1^ NAA. Rooted plants after both types of treatment (*in vitro* cultivation and thermotherapy) were then placed in a peat substrate with added Agriperlite. To confirm the success of GFLV inoculation and thermotherapy, RT-PCR testing for GFLV presence was carried out using standard procedures with primers described by MacKenzie *et al.* (1997).

DNA from the leaves of plants chosen for sampling was isolated using a DNeasy Plant mini kit (Qiagen), in accordance with manufacturer's instructions. The AFLP method was essentially as reported (Vos *et al.*, 1995) with minor modifications reflecting subsequent analysis with a ABI PRISM 310 genetic analyser (Applied Biosystems). The ligation mix was diluted 1/9 and 5 μL were added to the pre-amplification containing a 1 x reaction buffer, 100 ng of each of the primers *Eco*RI+A and *Mse*I+0, 12.5 nmol of each dNTP and 1 U DyNAzyme II DNA polymerase (Finnzymes), in a final volume of 50 μL. Pre-amplification was performed in a T-Gradient thermocycler (Biometra) as follows: 2 min at 94 °C; 20 cycles of 1 min at 94 °C, 1 min at 55 °C, 1 min at 72 °C and 10 min at 72 °C. The pre-amplification mix was diluted to a DNA concentration of 5 ng/ μL, whereupon 5 μL were added for selective amplification. For selective amplification, three differently labeled *Eco*RI+2 primers were mixed with one *Mse*I+2 primer in one single PCR reaction. Specifically, 5-FAM-5'-ACTGC GTACCAATTCAGG, JOE-5'-GACTGCGTACCAATTC ACT and NED-5'-GACTGCGTACCAATTCACC were used as fluorescent-labeled primers derived from the *Eco*RI recognition site, whereas 5'-GATGAGTCCTGAGTAAGC, 5'-GATGAGTCCTGAGTAAGC, 5'-GATGAGTCCTGA GTAAGC, 5'-GATGAGTCCTGAGTAAGC and 5'-GATGAGTCCTGAGTAAGC were used as primers derived form the *Mse*I recognition site. Selective amplification contained a 1 x reaction buffer, 30 ng of an *Mse*I- derived primer, 10 ng of individual *Eco*RI-derived primers, 3 nmol of each dNTP and 1.5 U of DyNAzyme II DNA polymerase in a final volume of 15 μL. Selective amplification was performed as follows: 13 touchdown cycles of 30 s at 94 °C, 30 s at 65 °C (-0.7 °C per cycle), 2 min at 72 °C; 22 cycles of 30 s at 94 °C, 30 s at 56 °C and 2 min at 72 °C, with a temperature ramp rate of 1 °C/s for all temperature changes. A total of 15 primer combinations were used.

Detailed manual evaluation was undertaken to ensure the highest possible accuracy in obtained spectra interpretation. Moreover, by overlapping the signals from all samples by using GeneScan software (Applied Biosystems), it was possible to evaluate each peak and its intensity in the context of the whole group of samples. A total of 1282 and 1521 AFLP amplicons were evaluated from the Müller Thurgau and Riesling groups of analysed variants, respectively.

The distribution of AFLP amplicons within individual analysed variants was classified as present (1) or absent (0), and was typed into a computer file as a binary matrix. The degree of similarity of the obtained qualitative data was calculated using the Nei and Li/Dice similarity index (Nei and Li, 1979). A dendrogram was constructed by means of the unweighted pair-group method using arithmetic averages (UPGMA method). Genetic similarity computing and the dendrograms were generated by NT-SYSpc software (version 2.11T, Exeter Software, USA).

The numbers of polymorphic loci found in individual somaclones are summarised in Table 2. The data show that some somaclones accumulated a significantly higher number of polymorphic loci. Similar results were also observed by Schellenbaum *et al.* (2008) and Li *et al.* (2007). The average polymorphism observed between *in vitro* cultivated somaclones was 32.5 (2.1%) and 21.5 (1.7%) for Riesling and Müller Thurgau variants, respectively, whereas somaclones after thermotherapy showed 32 (2.1%) and 17.3 (1.3%), respectively. Thus, in both groups of differently stressed variants, higher variability was encountered in the group of Riesling-derived somaclones. As to the impact of individual stress on the degree of detected polymorphism, no obvious general tendency was observed.

Concerning the similarity of obtained AFLP spectra, the average coefficients of similarity were 0.991 and 0.984 for Müller Thurgau and Riesling variants, respectively. In order to estimate the degree of accuracy in generating and evaluating AFLP spectra, the values obtained by the analysis of 3 identical variants were also calculated (if three different non-infected woody cuttings derived from one aboriginal maternal plant could be regarded as being identical). The average coefficient of similarity was 0.979 for both groups of identical variants derived from Müller Thurgau and Riesling. By comparing these with values obtained within the entire group of variants, it is possible to state that there were negligible changes in the DNA of plants exposed to the particular stress factors under investigation. The observed polymorphism could have been caused by real changes in the DNA, as well as unknown but probably significant arbitrary factors (AFLP artifacts, *Eco*RI sensitivity to C methylation if adjoined to its GAATTC recognition site, or discrepancies within peak evaluation).

The dendrograms in Figure 2 reflect the similarities of AFLP spectra obtained through the analysis of individual variants. In spite of the above mentioned caveats, it is possible to make a few interesting comments about the dendrograms:

a) The non-stressed maternal variants were most significantly detached within both dendrograms (MT-1-M, MT-2-M, R-1-M, R-2-M)

b) In the case of the Riesling dendrogram, the group of variants derived from maternal plant no. 15 was ordered within a single separated cluster

c) No tendencies in the ordering of variants on the base of their state of virus infection were recorded. Very weak tendencies in the clustering of variants on the basis of *in vitro* multiplication or thermotherapy were recognizable, especially in the case of Müller Thurgau variants.

The frequency of *in vitro* induced somaclonal variation is determined by a number of factors including genotype, explant source, duration of culture and medium composition (Skirvin *et al.*, 1994; Duncan, 1997; Brar and Jain, 1998). However, there have been no previous studies on the effects of stress induced by virus infections. Nevertheless, one could imagine that factors such as cultivar, virus disease and duration of infection could affect any potential changes in the genomes of host plants. Furthermore, a higher sensitivity to environmental stress due to the lower vitality of infected plants should not be over-looked.

In the case of grapevines, there are few, previously published studies focused on monitoring changes in plants that have undergone *in vitro* manipulation or mutagen action. Most of these monitored morphological and anatomical changes of *in vitro* plants. For example, Grenan (1984) described the morphological changes of leaves in plants originated *in vitro*. Dami and Hughes (1995) showed that leaves from *in vitro* cultivated plants lack normal palissade layers, contain larger mesophyll cells, have greater intercellular pore spaces and there are fewer chloroplasts than in those from greenhouse-grown plants. Gribaudo *et al.* (2000) compared the characteristics of self-rooted grapevine plants produced by micropropagation and those from woody cuttings. After some years in the vineyard, most of the observed parameters (phenology, vegetative growth, ampelography, production and juice composition) did not differ significantly. The only exception was the higher yield and different shape of leaves in the case of plants produced by micropropagation. Desperrier *et al.* (2003) obtained the somaclones of a variety of Gamay vine via somatic embryos produced by the *in vitro* culture of nucelli. Ten years of testing showed that the somaclones bore the same number of bunches of grapes, although they and their berries were smaller, thus giving rise to a drop in yield, often around 50%, in comparison with the control. This was expressed by the sharp increase in sugar content causing improved maturity of the somaclones.

**Figure 2:**
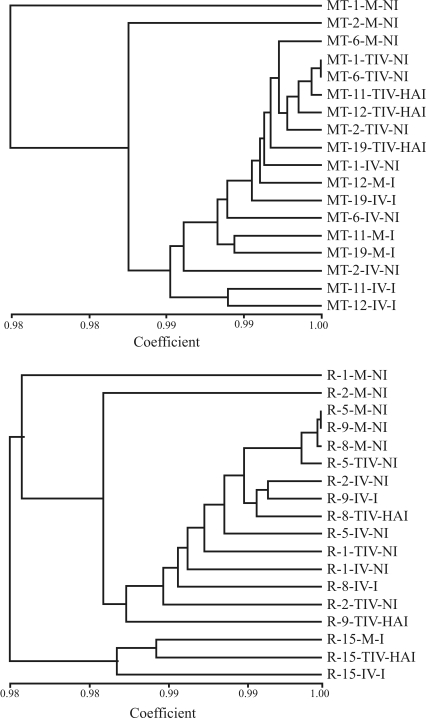
Dendrograms showing the similarity of AFLP profiles among analysed variants. Dendrograms were constructed based on Nei and Li/Dice similarity index and UPGMA method for clustering. Within a genetically similar group of variants, such as in this case it is probable that even a small proportion of non-genetic factors (peak evaluation, random occurrence of artifacts) could strongly influence the resulting clustering. Therefore the organisation of variants into the dendrograms shown here should be treated with certain caution.

In the case of cytological expertise, Kuksova *et al.* (1997) discovered that 2.5% of the plants regenerated from leaf explants through somatic embryogenesis were tetraploids (2n = 4x = 76). Gamma-irradiation (5-100 Gy) increased the frequency of tetraploid plant formation of primary (7%) and embryogenic calluses (7.6%). Some aneuploid plants were also found. Variability among regenerated plants, such as increased resistance to *Botrytis cinerea* and *Plasmopara viticola*, was also noted after field testing. Leal *et al.* (2006) employed flow cytometry to determine the ploidy level of *Vitis vinifera* L. somatic embryo-derived plants obtained by anther culture. Only one among the 41 analysed plants (2.4%) represented somaclonal variation (tetraploidy), all the others being diploid.

The genetic basis of the observed changes arising from stress is still poorly understood, only a few studies dealing with grapevines having been published so far. Harding *et al.* (1996) demonstrated that significant changes in DNA methylation can occur during the *in vitro* maintenance of grapevine shoot and callus cultures. The rDNA profiles of micropropagated subcultures showed that the percentage of recognition sequences containing a methylated external cytosine increased from 7.7% in glasshouse-grown plants to 64.5% for the first and 72.5% for the fourth micropropagated generation. Popescu *et al.* (2002) used AFLP techniques to describe the genetic variation in grapevines regenerated by anther culture. By using standard AFLP, the average coefficient of similarity among somaclones was determined as 0.8 in the case of plants obtained by direct embryogenesis, whereas polymorphism was absent in plants originating from indirect embryogenesis. Using MS-AFLP, polymorphism was detected in the case of both analysed groups. A higher degree of detected polymorphism was observed in the case of somaclones originating from indirect embryogenesis. By using five *Eco*RI/*Msp*I primer combinations, Schellenbaum *et al.* (2008) tested 56 ‘Chardonnay 96' and 22 ‘Syrah 174' somaclones derived from somatic embryos and compared them to their mother clones. They found that the percentage of polymorphic loci among individual somaclones varied from 0.0% to 3.2%, with the majority (48/78) exhibiting less than 0.8%. Nevertheless, it is necessary to remember that *Msp*I used for AFLP by Schellenbaum *et al.* (2008) shows sensitivity to DNA template methylation (*Msp*I will not cut if the external cytosine in CCGG loci is fully- or hemi-methylated - McClelland *et al.* 1994), hence probably affecting the degree of polymorphism they observed. Very high genetic variation frequency (9.3%) was found by Li *et al.* (2007) when using AFLP, where somaclones of wild barley were obtained by long-term tissue-culture regeneration from young, inflorescence-derived calli.

Thus, it can be deduced from the above mentioned publications that by using more extreme, somaclonal variation inducing conditions, we might observe a higher degree of genetic changes. However, our aim was to evaluate potential changes under conditions copying as closely as possible the prevailing situation in commercial breeding. As revealed by our AFLP analysis, genetic changes at the level of the primary structure of the DNA are minimal, if they occur at all. Furthermore, on the basis of the distribution of variants within the resulting dendrograms, there is no evidence to indicate that any of the three stress factors under observation had a significant influence on the degree of changes.

Thus, as revealed by our AFLP analysis, it seems that the unique and long-established properties of grapevines are not endangered by the possibility of changes in their DNA primary structure arising from applied stresses. On the other hand, there are still phenotypic or morphological changes in the case of *in vitro* cultivated plants. So, in order to obtain a more global view on this topic, our future studies will be focused on studying the epigenetic and transcriptome changes within the same groups of variants.

## Figures and Tables

**Table 1:** Groups of variants analysed by AFLP.

Cultivar	List of analysed mother plants	List of analysed *in-vitro* plants	List of analysed plants after thermotherapy
	R-1-M-NI	R-1-IV-NI	R-1-TIV-NI
	R-2-M-NI	R-2-IV-NI	R-2-TIV-NI
Riesling variants	R-5-M-NI	R-5-IV-NI	R-5-TIV-NI
	R-8-M-I	R-8-IV-I	R-8-TIV-HAI
	R-9-M-I	R-9-IV-I	R-9-TIV-HAI
	R-15-M-I	R-15-IV-I	R-15-TIV-HAI

	MT-1-M-NI	MT-1-IV-NI	MT-1-TIV-NI
	MT-2-M-NI	MT-2-IV-NI	MT-2-TIV-NI
Müller-Thurgau variants	MT-6-M-NI	MT-6-IV-NI	MT-6-TIV-NI
	MT-11-M-I	MT-11-IV-I	MT-11-TIV-HAI
	MT-12-M-I	MT-12-IV-I	MT-12-TIV-HAI
	MT-19-M-I	MT-19-IV-I	MT-19-TIV-HAI

System used for identifying variants: An identification number comes after the cultivar abbreviation (MT = Müller Thurgau, R = Riesling). The suffix M-NI means mother, non infected plant; M-I = mother, infected plant; IV-NI = *in vitro*, non-infected plant; IV-I = *in vitro*, infected plant; TIV - NI = *in vitro* thermotherapy, non-infected plant; TIV-HAI = *in vitro* thermotherapy, healthy plant after GFLV infection.

**Table 2:** Occurrence of polymorphic loci recognised in individual somaclones.

Type of somaclone	Registered polymorphic loci based on comparison with respective maternal clone (% of all evaluated loci)						Average for type of somaclone (%)
	R-1 M-NI	R-2 M-NI	R-5 M-NI	R-8 M-I	R-9 M-I	R-15 M-I	
*In vitro* cultivated	44 (2.9%)	36 (2.4%)	26 (1.7%)	34 (2.2%)	19 (1.2%)	36 (2.4%)	32.5 (2.1%)
*In vitro* thermo-therapy	47 (3.1%)	45 (3.0%)	10 (0.7%)	21 (1.4%)	38 (2.5%)	31 (2.0%)	32 (2.1%)

	MT-1 M-NI	MT-2 M-NI	MT6 M-NI	MT-11 M-I	MT-12 M-I	MT-19 M-I	
*In vitro* cultivated	35 (2.7%)	29 (2.3%)	14 (1.1%)	23 (1.8%)	17 (1.3%)	11 (0.9%)	21.5 (1.7%)
*In vitro* thermo-therapy	35 (2.7%)	20 (1.6%)	6 (0.05%)	13 (1.0%)	18 (1.4%)	12 (0.09%)	17.3 (1.3%)
